# Influence of Fiber Diameter of Polycaprolactone Nanofibrous Materials on Biofilm Formation and Retention of Bacterial Cells

**DOI:** 10.1021/acsami.4c03642

**Published:** 2024-05-08

**Authors:** Simona Lencova, Marta Stindlova, Kristyna Havlickova, Vera Jencova, Vaclav Peroutka, Katerina Navratilova, Kamila Zdenkova, Hana Stiborova, Sarka Hauzerova, Eva Kuzelova Kostakova, Ondrej Jankovsky, Pavel Kejzlar, David Lukas, Katerina Demnerova

**Affiliations:** † Department of Biochemistry and Microbiology, University of Chemistry and Technology, 160 00 Prague, Czech Republic; ‡ Department of Chemistry, Faculty of Science, Humanities and Education, Technical University of Liberec, 461 17 Liberec, Czech Republic; § Department of Inorganic Chemistry, University of Chemistry and Technology, 160 00 Prague Czech Republic; ∥ Department of Advanced Materials, Institute for Nanomaterials, Advanced Technologies and Innovation, Technical University of Liberec, 461 17 Liberec, Czech Republic

**Keywords:** nanofibers, polycaprolactone, morphology, biofilm, retention, bacteria

## Abstract

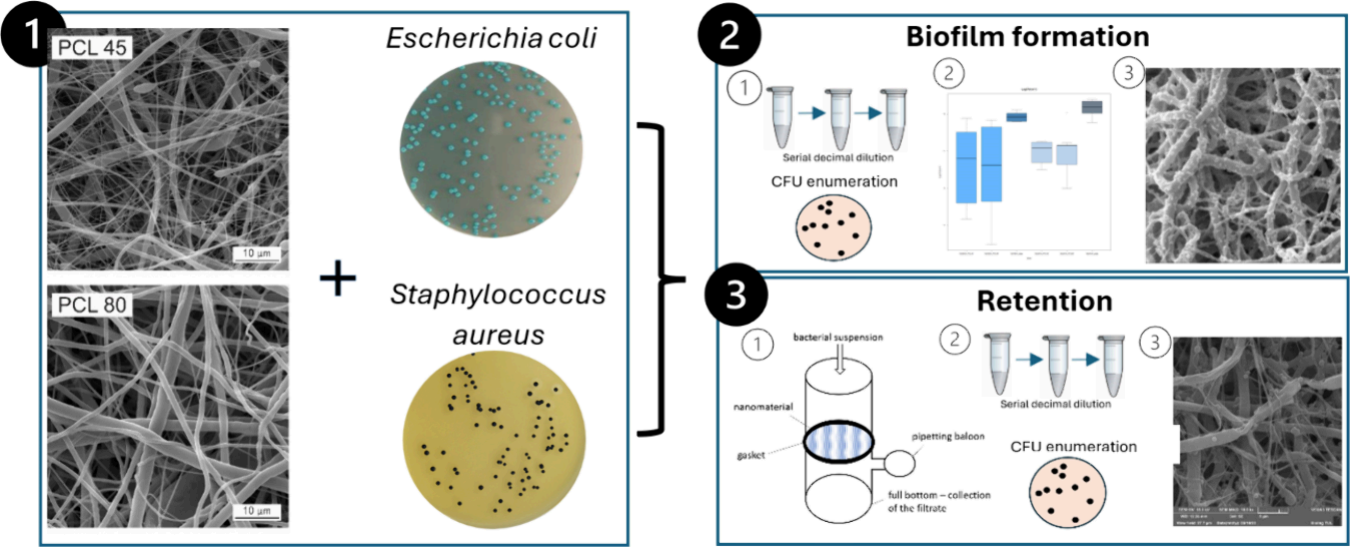

To develop microbiologically safe nanofibrous materials, it is crucial to understand their interactions with microbial cells. Current research indicates that the morphology of nanofibers, particularly the diameter of the fibers, may play a significant role in biofilm formation and retention. However, it has not yet been determined how the fiber diameter of poly-ε-caprolactone (PCL), one of the most widely used biopolymers, affects these microbial interactions. In this study, two nanofibrous materials electrospun from PCL (PCL45 and PCL80) with different fiber diameter and characteristic distance δ between fibers were compared in terms of their ability to support or inhibit bacterial biofilm formation and retain bacterial cells. Strains of *Escherichia coli* (ATCC 25922 and ATCC 8739) and *Staphylococcus aureus* (ATCC 25923 and ATCC 6538) were used as model bacteria. Biofilm formation rate and retention varied significantly between the *E. coli* and *S. aureus* strains (*p* < 0.05) for the tested nanomaterials. In general, PCL showed a lower tendency to be colonized by the tested bacteria compared to the control material (polystyrene). Fiber diameter did not influence the biofilm formation rate of *S. aureus* strains and *E. coli* 25922 (*p* > 0.05), but it did significantly impact the biofilm formation rate of *E. coli* 8739 and biofilm morphology formed by all of the tested bacterial strains. In PCL45, thick uniform biofilm layers were formed preferably on the surface, while in PCL80 smaller clusters formed preferably inside the structure. Further, fiber diameter significantly influenced the retention of bacterial cells of all the tested strains (*p* < 0.001). PCL45, with thin fibers (average fiber diameter of 376 nm), retained up to 7 log (CFU mL^–1^) of staphylococcal cells (100% retention). The overall results indicate PCL45’s potential for further research and highlight the nanofibers’ morphology influence on bacterial interactions and differences in bacterial strains’ behavior in the presence of nanomaterials.

## 1 Introduction

Electrospun nanofibrous materials are used widely in many spheres of human life, including medicine, the food industry, and biotechnology, and have been extensively studied since their invention. The research focuses on the safety of these nanomaterials, considering both their chemical–physical (such as toxicity and particle release) and microbiological aspects. As microorganisms can attach and colonize most polymeric nanomaterials commonly used for the production of wound dressings, food packaging, and biotechnology devices, their microbial contamination can lead to serious health risks and economic losses.^1,2^

Nanomaterials are often modified with antimicrobial substances, like antibiotics, to ensure microbiological safety by suppressing microbial growth.^3^ However, due to the overuse and misuse of antibiotics leading to increasing threat of the emergence and spread of antibiotic resistance, new approaches to achieve microbiological safety without antibiotics are being intensively sought.^4^

Recent research indicates that besides nanofibers’ modification with antimicrobials, morphological parameters can reduce microbiological risks by affecting interactions between nanofibers and microorganisms. Specifically, fiber diameter and surface density are considered the main factors influencing microbial attachment, colonization, biofilm formation, and the nanomaterial’s ability to retain microbial cells during filtration.^5−8^

In the past decade, several studies have examined various polymeric nanofibrous materials. Kargar et al.^6^ and Abrigo et al.^5^ investigated the influence of polystyrene (PS) nanofibers’ fiber diameter on bacterial colonization. Kargar et al. concluded that *Pseudomonas aeruginosa* adhesion increased with increasing fiber diameter; the study was conducted on materials with fiber diameter 70–1100 nm.^6^ Abrigo et al. confirmed that the fiber diameter influenced the adhesion and proliferation of *P. aeruginosa*, *Escherichia coli*, and *Staphylococcus aureus* on PS nanofibers with diameters of 500 and 3000 nm. Abrigo et al. highlighted that fiber diameter similar to bacterial cell size supported adhesion and proliferation of bacterial cells the most.^5^

Further, the influence of polyamide (PA) nanofibers’ fiber diameter (87–236 nm) and surface density on biofilm formation by *E. coli*, *S. aureus*, and *Staphylococcus epidermidis* was investigated in the study of Lencova et al.^7^ It was verified that fiber diameter influenced biofilm formation; less biofilm formed on nanomaterials with (i) thicker fibers and high surface density and (ii) thin fibers with low surface density. Generally, less biofilm formed on nanomaterials with fibers either closely or widely spaced, preventing bacteria from forming clusters or bridges between fibers. Additionally, the nonfunctionalized PA nanomaterial most inhibiting biofilm formation from the tested ones was equally effective to the PA nanomaterial functionalized with 0.1 wt % of AgNO_3_.^7^ The same set of PA nanomaterials was further tested in terms of their retention capacity for *S. aureus* cells. Retention was influenced by surface density, while the effect of fiber diameter was not found.^8^

Unlike PS and PA, the conclusions of morphologically focused studies of poly-ε-caprolactone (PCL) fibers, commonly used in medical and biotechnological applications, are inconsistent. PCL microfibers and nanofibers differing in fiber diameter were investigated in the studies of Tamay-Ramos et al.^2^ and De Cesare et al.^1^ Tamayo-Ramos et al. found that PCL microfibers’ fiber diameter (2.2–6.4 μm) did not influence the loading capacity of *E. coli*, *Pseudomonas putida*, *Brevundimonas diminuta*, and *Sphingobium fuliginis*.^2^ In contrast, De Cesare et al. reported that the PCL fiber diameter affects microbial interactions. Specifically, *Burkholderia terricola* was attached preferably to PCL nanofibers with a fiber diameter of 100 nm, while thinner fibers were colonized less frequently.^1^

The above-mentioned studies highlight the need for further research focusing on the clarification of nanofiber morphology influence on microbial interactions. Emphasizing the urge to better understand this problem and to speed up the development of microbiologically safe PCL nanomaterials, this paper aims to deepen insight into the influence of PCL fiber diameter on biofilm formation and bacterial retention. The scope of this study ranges from the preparation and characterization of two PCL nanofibrous materials differing in fiber diameter and δ distance between fibers, via qualitative and quantitative analysis of the above-mentioned interactions with clinically relevant opportunistically pathogenic bacteria *E. coli* and *S. aureus*, to statistical evaluation of the results and drawing the recommendations for following research. We report that the fiber diameter of PCL influences bacterial interactions, mainly retention. Biofilm formation is affected in terms of the biofilm structure and preferred localization in the nanomaterial. The quantity of biofilm formation may be influenced by PCL fiber diameter, but we found varying results for specific bacterial strains, and no general statement can be made.

## 2 Materials and Methods

### 2.1 Nanomaterials Preparation

#### 2.1.1 Polymers for Nanofibers Preparation

Polymer PCL with an average molecular weight of *M*_n_ 45000 g mol^–1^ and PCL with an average molecular weight of *M*_n_ 80000 g mol^–1^ (both purchased from Merck KGaA, Germany) were used as the polymer materials for the preparation polymer solution and electrospinning process. The polymer materials were dissolved in the solution system chloroform/ethanol at a weight ratio of 8:2. Both solvents were purchased from Penta, Czechia. The final polymer concentration was 16 and 10 wt %. Details of polymer solutions are shown in Table 1.

**Table 1:** Characterization of the Polymer Solutions for the Electrospinning Process and the Process Conditions of Electrospinning

sample	polymer (*M*_n_)	solvent system	polymer concn (w/w)	withdrawal speed (mm min^–1^)	metal insert diam (mm)
PCL45	PCL (45000)	chloroform:ethanol 8:2 w/w	16	46	0.6
PCL80	PCL (80000)	chloroform:ethanol 8:2 w/w	10	20	0.7

#### 2.1.2 Electrospinning

The materials were electrospun from freshly prepared polymer solutions after 10 h of dissolving at a magnetic stirrer. Nanofibrous materials were DC electrospun on an NS 1WS500U Nanospider electrospinning machine (Elmarco, Czechia) with a stationary wire-spinning electrode. The electrospinning conditions were set according to previous optimizations. The spinning electrode was positively charged using a direct current high voltage source (40 kV), and the collector was negatively charged (−10 kV). The distance between the spinning electrode and collector was set at 185 mm. The polymer solution was fed onto the wire spinning electrode by using a moving carriage module filled with the polymer solution. The layer of the polymer solution on the spinning electrode was regulated by a metal insert. Stable conditions during the electrospinning process were ensured by an air conditioning unit. The temperature was set at 22 ± 2 °C and relative humidity at 50 ± 2%. The withdrawal speeds were changed for each polymer solution (shown in Table 1) to obtain similar final areal weights of both nanofibrous layers.

#### 2.1.3 Sterilization

Materials were sterilized by ethylene oxide at RT for 12 h in an Anprolene sterilizing device (model AN74, Andersen Sterilizer, United Kingdom). The ethylene oxide sterilization process was performed according to the ISO 11135-1 “Sterilization of products for health care sterilization with ethylene oxide - part 1: Requirements for the development, validation, and continuous control of the sterilization procedure for medical devices” standard. The materials were then vented at room temperature for more than 2 weeks.

### 2.2 Nanofibers Characterization

#### 2.2.1 Scanning Electron Microscopy

Scanning electron microscopy (SEM) was used for the morphological analysis of nanofibrous materials. Electrospun samples (5 × 5 mm^2^) were sputtered with a thin layer of gold and imaged by a Vega 3SB Easy Probe (TESCAN, Czechia) scanning electron microscope. The fiber diameters (100 measurements for each sample) were measured by using FIJI/ImageJ software (NIH). Further, a quantified estimation of characteristic distances between fibers forming “eyes” in SEM photographs of fiber systems was provided according to Lurie et al.^9^ We estimate here the characteristic intrafiber distance δ according to eq 1.
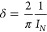
1Detailed information is provided in the Supporting Information. Next, SEM at low voltage to analyze the fiber surface was performed. The samples were sputtered with 1 nm of platinum for surface analysis. High-resolution imaging was performed on the FE-SEM Zeiss Ultra Plus equipped with the energy-dispersive spectroscopy (Oxford X-Max20, Carl Zeiss, Germany) using gentle beam conditions of 1 kV/1.6 pA.

#### 2.2.2 BET

The specific surface area was determined using Brunauer–Emmett–Teller (BET) analysis with nitrogen adsorption and desorption on the sample surfaces (Autosorb iQ-KR/MP, Quantachrome) according to Hauzerova et al.^10^

#### 2.2.3 X-ray Photoelectron Spectroscopy

X-ray photoelectron spectroscopy (XPS) was measured using an XPS SPECS spectrometer equipped with an XR 50 MF monochromatic X-ray radiation source (1486.7 eV) and a Phoibos 150 2D-CCD hemispherical analyzer and detector. The pressure inside the chamber during the experiments was 5 × 10^–10^ mbar or lower. Wide-scan surveys were performed with *E*_p_ = 80 eV, with subsequent high-resolution scans of the desired core lines with *E*_p_ = 50 eV. The samples were placed on a conductive carrier made from a high-purity gold plate deposited on silicon dioxide. All peaks were charge corrected to the adventitious C 1s peak at 284.6 eV. The CasaXPS program was used for analyzing the XPS spectra.

#### 2.2.4 Wettability

The wettability of PCL electrospun nanofibrous materials was tested by a Krüss K100 microtensiometer (Krüss GmbH, Germany). Dynamic wetting was tested by a Washburn adsorption test (wicking test). The sample of 30 mm width and 40 mm length was cut and placed into the holder for foils vertically. The holder with the nanofibrous sample was then inserted into the clip, with the bottom sample edge arranged in a horizontal position and parallel orientation of the sample edge during immersion in the test liquid (distill water, Aqual 29, Verkon, Brands nad Labem, Czech Republic). The ambient conditions were a temperature of 22 °C and 58% relative humidity for all sample measurements. The wicking weight of water wicked into the fibrous system in time into PCL45 and PCL 80 was measured five times (from five samples) for each material.

### 2.3 Bacterial Strains and Culture Conditions

Four bacterial strains were used in this study: Gram-positive bacteria *S. aureus* ATCC 25923 (eq CCM 3953; origin: clinical isolate) and *S. aureus* ATCC 6538 (eq CCM 4516; origin: human lesion) and Gram-negative bacteria *E. coli* ATCC 25922 (eq CCM 3954; origin: clinical isolate) and *E. coli* ATCC 8739 (eq CCM 4517; origin: human feces) obtained from the Czech Collection of Microorganisms (CCM, Czechia). The bacteria were stored in Tryptone Soy Broth (TSB, Oxoid Ltd., United Kingdom) mixed with 25% glycerol (Penta, Czechia) at −80 °C. Before each analysis, bacterial suspensions were inoculated into sterile TSB (5 mL) and incubated for 24 h at 37 °C.

### 2.4 Biofilm Formation

PCL nanomaterials of size 1 × 1 cm^2^ were sterilized. PCL nanomaterials prepared this way were used as platforms for single-species biofilm formation experiments by the above-mentioned bacterial strains and treated as follows. The defined pieces of PCL nanomaterials were placed in a 12-well microtiter plate (Greiner Bio-One, Austria) containing 3 mL of bacterial suspension (TSB, concentration approximately 10^6^ CFU mL^–1^). Three types of control wells contained (i) 3 mL of pure TSB as a control of medium sterility, (ii) 3 mL of the bacterial suspension for confirmation of bacteria growth, or (iii) 3 mL of pure TSB with a sterile piece of PCL nanomaterial as a control of sterility of the used nanomaterial. After cultivation (37 °C, 48 h), the determination of viable cells adhered to the nanomaterials and the control wells was provided as specified below.

### 2.5 Determination of Viable Cells Adhered to the Nanomaterials by CFU Enumeration

Biofilm formation on nanomaterials and the bottom of control wells was determined as described by Lencova et al.^7^ Briefly, nanomaterials with mature biofilms were washed five times with sterile distilled water. The washed nanomaterials were placed into a sterile 12-well plate and allowed to dry (45 min, room temperature). Sterile Mueller-Hinton Broth (MHB, Oxoid Ltd., United Kingdom) was added to the wells (1 mL of MHB per well). The plates were sonicated (3 min), and the obtained suspensions with released biofilm-forming cells were serially decimally diluted in physiological saline solution up to the eighth dilution. Twenty microliter droplets of particular dilutions were applied onto a Tryptone Soy Agar (TSA, Oxoid Ltd., United Kingdom) in three parallels and incubated (24 h, 37 °C). After the incubation, CFU were enumerated and quantified as described previously;^7^ CFU per 1 cm^2^ (CFU/cm^2^) of the nanomaterial was determined. The control wells containing pure TSB or only a bacterial suspension were treated similarly. The wells were washed five times with distilled water. After the cells were dried for 45 min at room temperature, 1 mL of MHB was added to each cell. The cells were released by sonication, and the obtained solutions were serially decimally diluted; 20 μL droplets were applied onto TSA and after cultivation (37 °C, 24 h); CFU/cm^2^ of the well and the biofilm formation rate (%) were determined.

### 2.6 Retention Assay

Filtration of particular bacterial suspensions was provided as described in Lencova et al.^8^ Briefly, 3 mL of the bacterial suspensions (1.0 and 0.5 McF, separately, to ensure a robust analysis) was filtrated through a sterile PCL membrane (diameter 5 cm). The filtrate was collected in the bottom part of the apparatus. After the filtration, the bacteria both in the filtered suspension and in the obtained filtrate were quantified as follows. Both suspension and filtrate were serially decimally diluted in physiological saline solution for up to an eighth dilution and processed as stated in Section 2.4. Then, log removal (CFU mL^–1^) and retention rate (%) were calculated as described previously.^8^

### 2.7 SEM Analysis

Samples of PCL nanomaterials, after both filtration and biofilm formation, were analyzed by SEM. The PCLs with the retained cells or matured biofilm were gently rinsed with phosphate-buffered saline (PBS) and fixed (4 °C, 15 min) in frozen ethanol (99.8%, Penta, Czechia). The samples were dewatered with ethanol (concentrations 60.0–99.9%; 5 min per each concentration, room temperature). After drying (room temperature, at least 24 h), the samples were sputter-coated with gold (14 nm) and observed using a SEM Tescan Vega3 SB Easy Probe (Tescan, Czechia).

### 2.8 Statistical Analysis and Tested Hypotheses

The experiments were performed in at least three biological replicates and three technical replicates. Data sets (after discarding outliers according to Dean–Dixon’s Q-test) were subjected to the Shapiro–Wilk test, a numerical test of significance comparing the distribution of the sample with a normal distribution and, at a given level of significance, determining whether or not the data show a significant deviation from the normal distribution. The data were considered normally distributed at *p* > 0.05 (Tables S1 and S2). Normally distributed data were accordingly subjected to the paired *t* test, unpaired two-sample *t* test, and multiple comparisons by one-way analysis of variance (ANOVA). The difference was assumed to be significant at *p* < 0.001, *p* < 0.01 and *p* < 0.05. If the ANOVA result was significant, Tukey HSD (Tukey Honest Significant Differences) was calculated to perform multiple pairwise comparisons of groups’ means. Statistical analysis was performed using the R programming language in the RStudio program and was used for evaluation of the following hypotheses focusing on the influence of PCL fiber diameter on (i) bacterial single-species biofilm formation (H1, H2) and (ii) retention of bacterial cells (H3, H4):

H1: PCL nanomaterials’ fiber diameter of PCL nanomaterials influences bacterial biofilm formation.

H2: Biofilm formation by *Staphylococcus* and *Escherichia* on PCL nanomaterials differs.

H3: PCL nanomaterials’ fiber diameter of PCL nanomaterials influences the retention of bacterial cells.

H4: PCLs’ retention of *Staphylococcus* and *Escherichia* cells varies.

## 3 Results and Discussion

### 3.1 Nanomaterials Preparation and Characterization

The materials considered in the present study were prepared from PCL of different molecular weights (45000 and 80000) to create morphologically diverse fibrous layers, which were subjected to experiments examining the interaction with microorganisms. The analysis of PCL nanomaterials surface was performed by SEM analysis (conventional and in low voltage) and XPS analysis, as these methods are considered the most suitable.^11^ The analysis was further supplemented by BET and wettability measurement.

**Figure 1:**
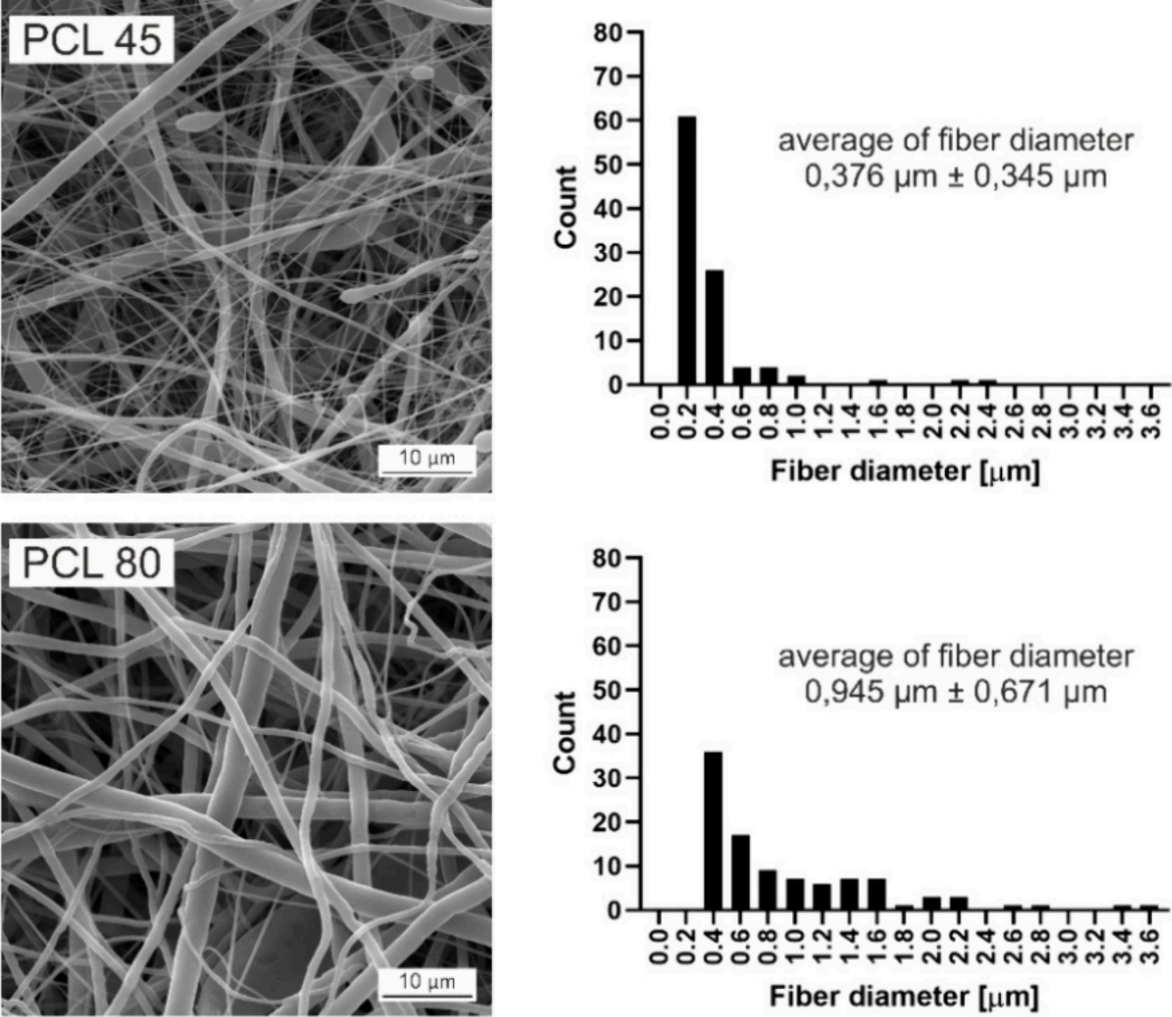
SEM images of the PCL45 and PCL80 nanofibrous material supplemented by histograms showing the distribution of the fiber’s diameter.

**Figure 2:**
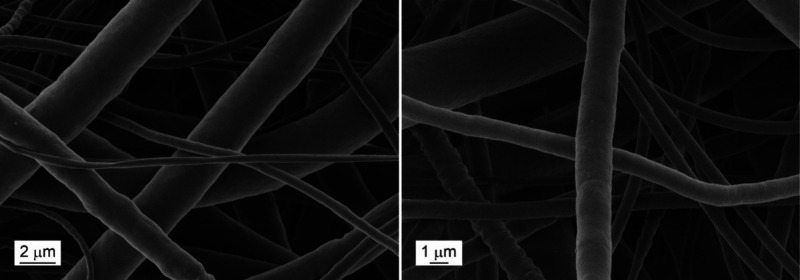
SEM images at low voltage for the analysis of PCL80 fibers surface.

The SEM analysis proved that the morphologies of the resulting nano/microfiber layers are homogeneous without significant visible defects (Figure 1). The SEM images were complemented by histograms depicting the diameters of the electrospun fibers. The findings revealed a significant impact of the polymer’s molecular weight on the diameter of the electrospun fibers. Maintaining consistent process conditions and a comparable viscosity of polymer solutions during electrospinning resulted in larger diameter fibers for higher molecular weights. This correlation is evident in the SEM images presented in Figure 1 along with the corresponding average fiber diameter values detailed in Table 2. As the fiber diameter decreased, the specific surface area, measured through BET analysis, increased. The specific surface area determined for PCL45 (fiber diameter 0.376 ± 0.345 μm) was 1.82, while for PCL80 (fiber diameter 0.945 ± 0.671 μm) was 1.40.^10^ Further, the characteristic distance δ between fibers was determined as a complementary analysis specifying nanomaterials structure. The characteristic distance δ in the nanofibrous sample PCL80 was estimated as 1.07 ± 0.08 and as 0.56 ± 0.1 μm for the sample PCL45 (Supporting Information). The morphology of the fibers and the ability to tune the structure according to requirements, especially the diameter of the fibers and the specific surface play a crucial role in their ability to influence or inhibit microbial growth.^12−14^ As a complementary analysis, SEM at a low voltage was performed. The observed surface of the PCL fibers was smooth, with no specific discrepancies (Figure 2). With regard to materials wettability, no significant differences between the two tested materials were found (Figure S1).

**Table 2:** Characterization of the Electrospun Materials

sample	areal weight (g m^–2^)	fiber diameter (μm)	distances between fibers (μm)	specific surface area (m^2^ g^–1^)
PCL45	20.6 ± 1.1	0.376 ± 0.345	0.56 ± 0.11	1.82
PCL80	20.0 ± 1.4	0.945 ± 0.671	1.08 ± 0.08	1.40

**Figure 3:**
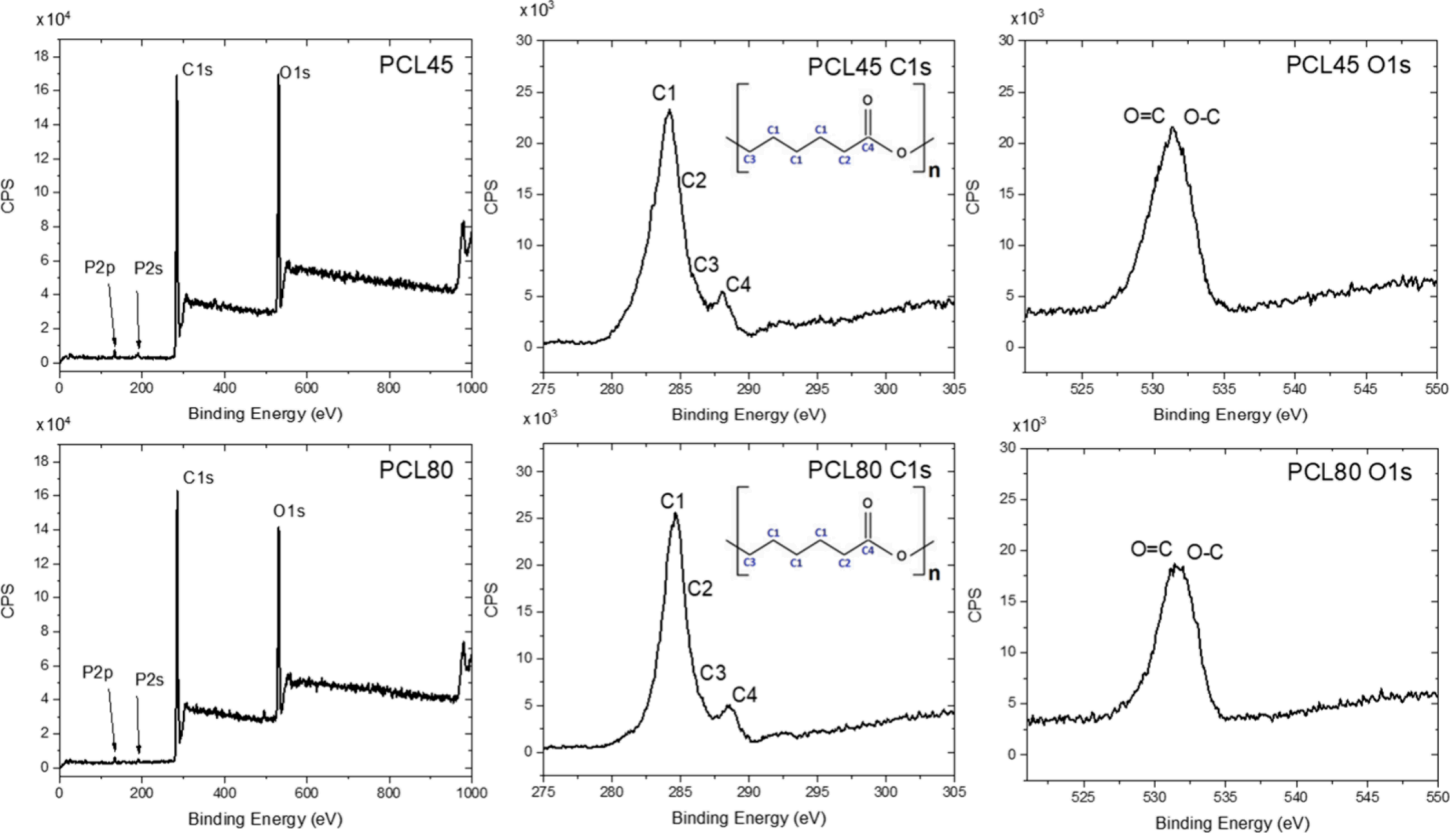
XPS survey spectra and C 1s and O 1s details for PCL45 and PCL80.

Samples were further analyzed by X-ray photoelectron spectroscopy (XPS). The XPS survey spectra as well as C 1s and O 1s details are shown in Figure 3. From the survey spectra, the C/O ratio (at.) was 1.1/1 for PCL45 and 1.3/1 for PCL80. Except for carbon and oxygen, also traces of phosphorus were detected for both samples (see P 2p and P 2s at ∼133 and ∼190 eV). Details of the C 1s band showed the following carbon states: C–C (C1), C*–C=O(−O) (C2), C–O (C3), and C=O(−O) (C4), while in O 1s the presence of O=C and O–C was confirmed, which is in good agreement with the literature.^15^

PCL was selected as an appropriate polymer for this work due to its properties and wide range of applications. PCL is a synthetic biodegradable polymer, which is used as a biocompatible material for the development of scaffolds in tissue engineering, wound dressings, face masks, drug-delivery systems, and food packaging.^16^ PCL and other nanofibrous materials can serve as a key element in the fight against microbial infections, and their use is becoming increasingly important in the development of antibacterial applications. The nano/microfibrous nature of these materials provides a structure very similar to tissue extracellular matrix (ECM), in particular a high surface area to volume ratio of the material, a high degree of porosity, and tunable pore size, which allows the materials to create an environment similar to native tissues, thereby promoting cell adhesion and proliferation, making the materials promising candidates for tissue engineering applications.^17,18^ In the case of microorganisms, the unique structure of the materials can achieve a protective effect, and nano/microfibrous materials can act as a protective barrier to minimize the risk of bacterial infection or reduce its effectiveness.^3^ Following this assumption and the thorough characterization of the prepared PCL materials, we investigated bacterial interactions with the PCLs were investigated.

### 3.2 Biofilm Formation

**Figure 4:**
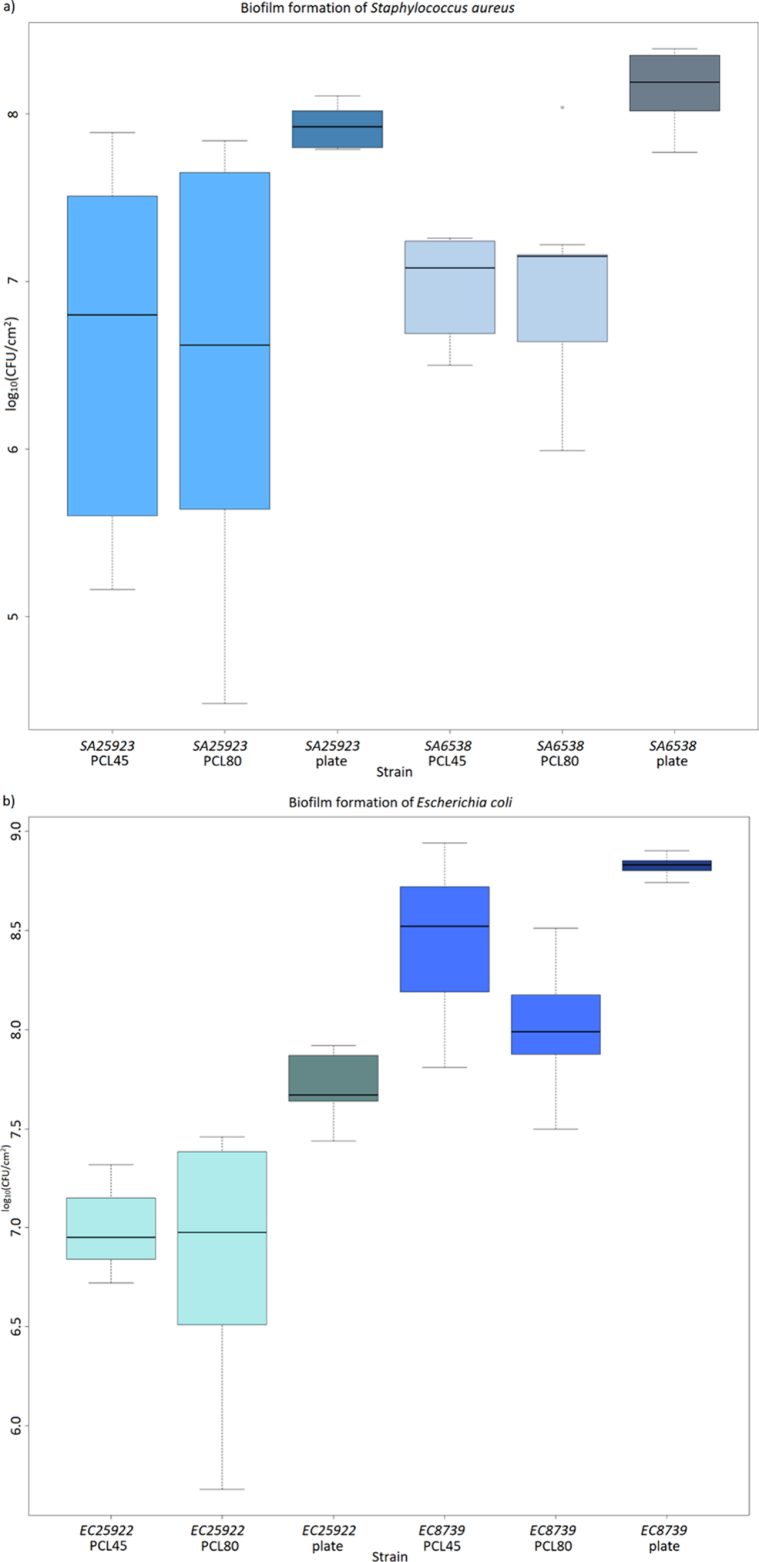
Biofilm formation by (a) *S. aureus* 6538 (SA6538) and *S. aureus* 25923 (SA25923) and (b) *E. coli* 8739 (EC8739) and *E. coli* 25922 (EC25922) on PCL45 and PCL80 and control (plate, PS) expressed as the determination of CFU/cm^2^.

**Figure 5:**
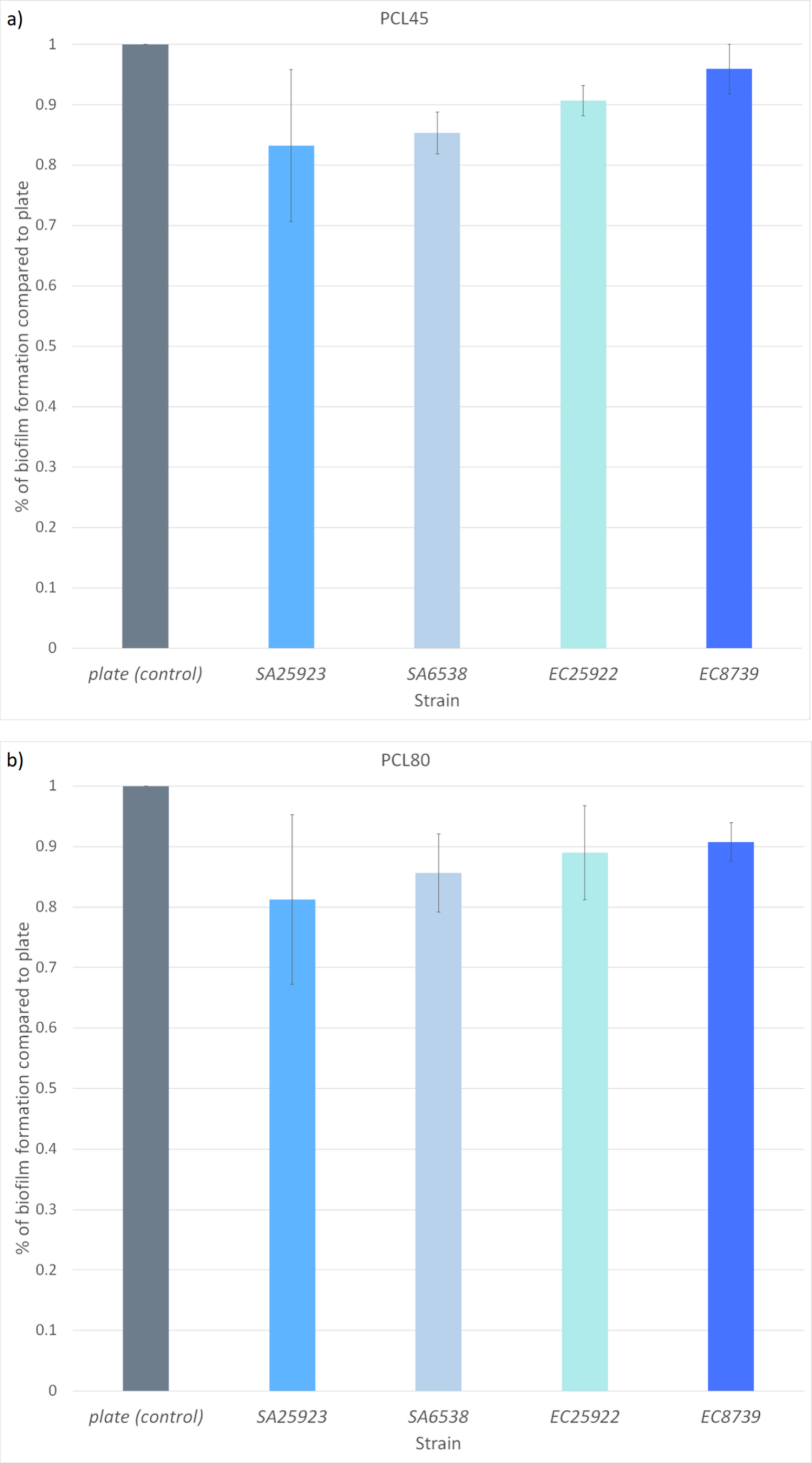
Biofilm formation by *S. aureus* 6538 (SA6538), *S. aureus* 25923 (SA25923), *E. coli* 8739 (EC8739), and *E. coli* 25922 (EC25922) on PCL45 (a) and PCL80 (b) and control (plate, PS) expressed as biofilm formation rate compared to control (%).

Biofilm formation by four bacterial strains, representing clinically, biotechnologically, and food-relevant bacteria, on PCL nanomaterials, and a control material, PS, was assessed through both quantitative and qualitative analyses. The quantitative evaluation involved determining CFU/cm^2^ and biofilm formation rate (Figures 4 and 5, Table S3), with all data subjected to statistical analysis (Tables S1 and S4–S7). The qualitative assessment was conducted using SEM (Figure 5). As model organisms, we used two strains of *E. coli* and two strains of *S. aureus*, representing clinically, biotechnologically, and food-relevant bacteria.

Biofilms, defined as microbial cell gatherings irreversibly associated with a surface,^7^ formed on both tested PCL nanomaterials, which aligns with the expectations published studies indicating that nonfunctionalized PCL materials are typically easily colonized with bacteria.^1,2^ Determined biofilm amounts were significantly higher on PS than PCL nanomaterials (*p* < 0.01); biofilm formation on PCLs reached 8.5–61.3% of biofilm formation rate compared to PS in average (Figure 4, Tables S3 and S4).

Despite the lower biofilm formation rate on PCLs compared to PS, still, very dense biofilms consisting of 1.1 × 10^7^–3.9 × 10^8^ CFU/cm^2^ on average were assessed on PCLs. This ease with which bacterial biofilms were formed on PCLs predestinates their potential for microbial biomass production in biotechnological applications,^16^ while also highlighting the importance of controlling the microbial quality of PCL materials applicable in medicine and the food industry. For all these practical implementations, knowledge of how PCL’s fiber diameter influences microbial cell adhesion and biofilm maturation is fundamental; this would allow us to control biofilm formation. The biomass amount would be purposefully increased for biotechnology-relevant microorganisms, while material colonization by pathogens could be reduced for medically and food usable materials.

**Figure 6:**
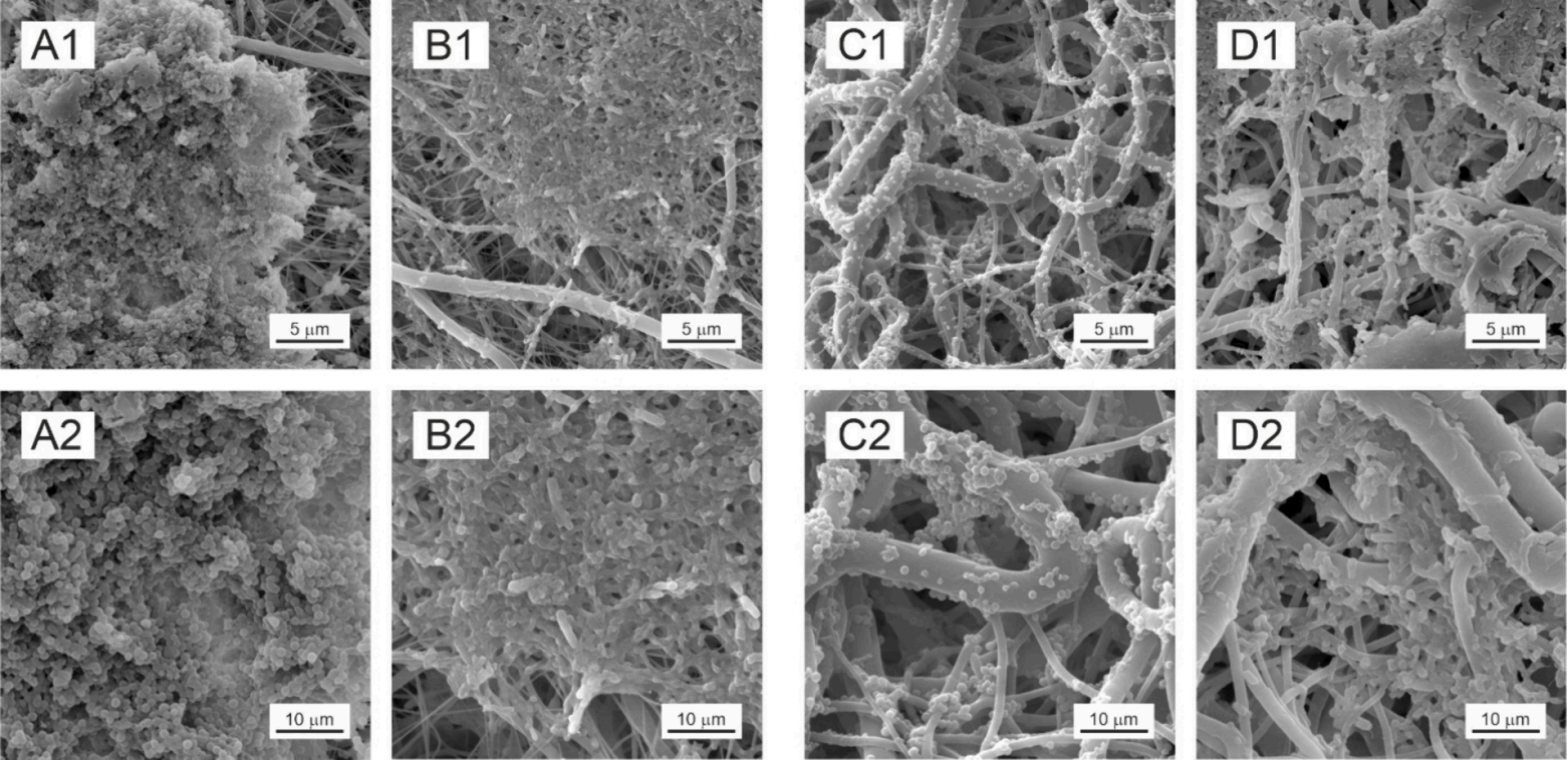
Biofilm formation on PCL nanomaterials visualized by SEM; biofilm formed on PCL45 by *S. aureus* 6538 (A1, A2) and *E. coli* 8739 (B1, B2) and on PCL80 by *S. aureus* 25923 (C1, C2) and *E. coli* 25922 (D1, D2).

Realizing that and taking into account not uniform conclusions in this field,^1,2^ we focused on evaluating how PCL’s fiber diameter affects biofilm formation and draw the initial hypothesis H1: “PCL nanomaterials’ fiber diameter influences bacterial biofilm formation”. While biofilm amounts slightly varied among tested PCL materials and strains (Figures 4, 5, and 6), statistical significance between biofilms formed on PCL45 and PCL80 was confirmed only for strain *E. coli* 8739 (*p* < 0.05), while no significant difference was found for *S. aureus* 25923, *S. aureus* 6538, and *E. coli* 25922 (*p* > 0.05) (Table S5). Based on that H1 was confirmed for only one of the tested strains, *E. coli* 8739, while it was rejected for *S. aureus* strains and *E. coli* 25922.

Although the biofilm biomass formed on PCL45 and PCL80 did not differ for three of the tested bacterial strains, the biofilm structure and localization varied between tested PCLs for all four tested bacterial strains (Figure 6). In PCL45, thick uniform biofilm layers were formed preferably on the surface, while in PCL80 smaller clusters formed preferably inside the structure. That indicates that bacterial biofilms form preferably in PCL locations, where there is enough space to attach and further expand. As PCL45 dispones with thin fiber, the gaps between them are small, and tested bacterial strains prefer PCL45’s surface. Oppositely, in PCL80 with thicker fibers, larger gaps between single fibers can be seen and bacteria prefer to colonize these inner structures of the nanomaterial. We consider this phenomenon important for applications, as materials colonized on their surface can be easily sterilized by common methods than materials colonized inside their structure.

Following these findings, H2: “Biofilm formation by *Staphylococcus* and *Escherichia* on PCL nanomaterials differs.” was tested. The average biofilm formation of *S. aureus* was determined as 19.2 ± 15.0% on PCL45 and 25.4 ± 17.4% on PCL80; the average biofilm formation of *E. coli* was determined as 40.4 ± 29.5% on PCL45 and 18.7 ± 1.5% on PCL80 (Figure 5 and Table S3). H2 was confirmed for PCL45 (*p* < 0.01), on which *E. coli* biofilms were significantly denser, while was declined for PCL80 (*p* > 0.05), on which *Staphylococcus* and *Escherichia* biofilms formed equally. Further, we investigated whether biofilm formation on the PCL nanomaterial varies among strains of the same species. Based on statistical analysis, no difference was found for *S. aureus* strains (*p* > 0.05), while confirmed for *E. coli* strains (*p* < 0.01).

These results highlight the need for further research in this field, emphasizing the importance of testing at least two strains of specific bacterial species in all of the studies. It is well-known that various species and even strains of the same species behave differently, as exemplified in our study with two distinct *E. coli* strains originating from different sources. This phenomenon has been documented in recent studies, where differences in genetic apparatus, influenced also by the strains’ origin, are usually identified as one of the main factors.^19^

Various characteristics and behavior of different bacterial strains along with very different fiber sizes tested could be the possible reason for the contradictions in the conclusions of studies focusing on the influence of the PCLs’ fiber diameter on biofilm formation. Tamayo-Ramos et al. studied the biofilm formation of *E. coli*, *P. putida*, *B. diminuta*, and *S. fuliginis* (one strain of each species) on PCL microfibers with fiber diameter ranging between 2.2 and 6.4 μm and concluded that fiber diameter had no significant influence;^2^ De Cesare et al. studied biofilm formation of one strain of *B. terricola* on PCL nanofibers with fiber diameter smaller than 100 nm and concluded that thicker fibers (with fiber diameter approximately 100 nm) supported attachment and biofilm formation more than thin ones.^1^ In our study, we aimed to fill the gap between tested fiber diameters; thus, we selected PCLs with fiber diameter between the diameters tested in the above-mentioned studies, specifically 376–976 nm, and bacteria *E. coli* and *S. aureus*.

Although the results mostly support the assumption that PCLs’ fiber diameter does not influence biofilm formation, the results are not uniform for all the tested bacterial strains, and no general conclusion on PCLs’ fiber diameter effect on biofilm formation can be made. Thus, we emphasize the need for testing of individual bacterial species and strains for specific practical applications of the particular PCLs and further detailed research including a wide range of tested PCLs’ fiber diameters and bacterial strains. These results further point to differences in the influence of the morphology of nanomaterials electrospun from different polymers on microbial interactions. In contrast to PS and PA nanomaterials, for which fiber diameter influence on biofilm formation was confirmed in several studies,^5−7^ PCL nanomaterials remain unclear as the conclusions of studies focusing on PCL fiber diameter influence on biofilm formation are significantly different.^1,2^

To summarize the results, the following conclusions were made: (i) Biofilms form easily on PCL nanomaterials but less than on PS. (ii) The fiber diameter of PCL nanomaterials may influence the biofilm formation rate, and no general conclusion can be made; it depends on particular bacterial strains. (iii) The fiber diameter influences morphology and localization of biofilm formed on nanomaterials. (iii) The biofilm formation rate on PCL nanomaterials differs among bacterial species, *E. coli* and *S. aureus*, and may vary among various strains of the same species.

### 3.3 Retention of Bacterial Cells

**Figure 7:**
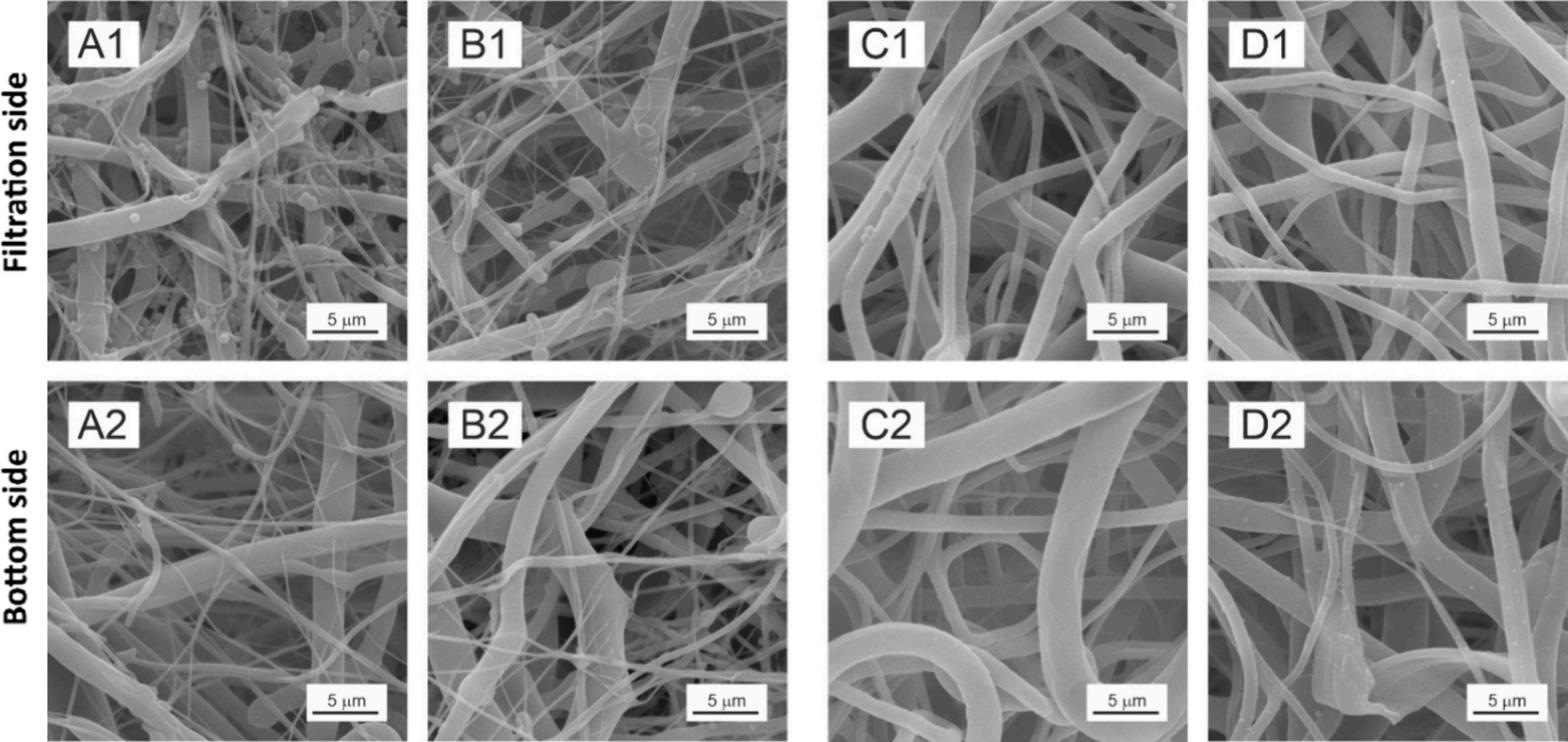
PCL45 after the filtration of *S. aureus* 6538 (A1, A2) and *E. coli* 8739 (B1, B2) and PCL80 after the filtration of *S. aureus* 25923 C1, C2) and *E. coli* 25922 (D1, D2) visualized by SEM. For each sample, the filtration side (through which the suspension was filtrated) and bottom side are displayed.

**Figure 8:**
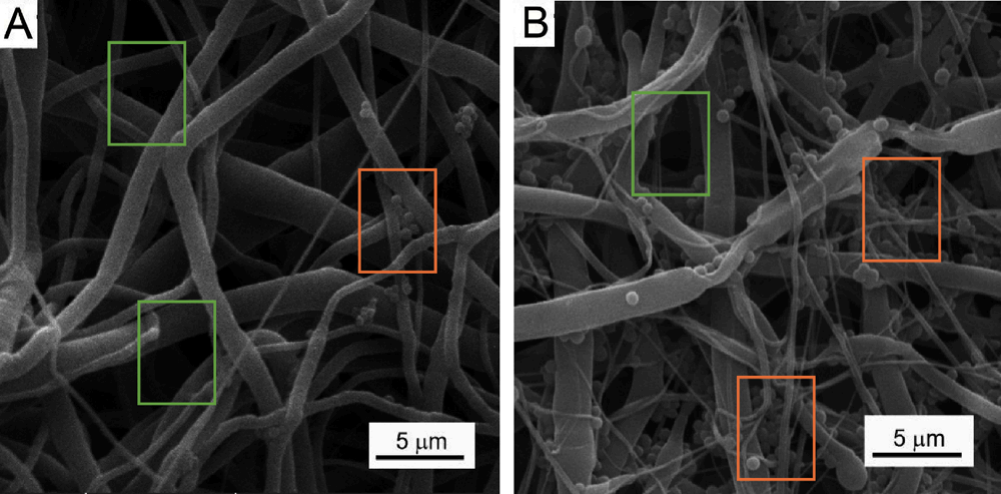
Effect of fiber diameter on bacterial cell retention; bacteria are retained in a tangle of small diameter fibers (highlighted in an orange rectangle), while no retention occurs in areas with predominantly strong fibers (highlighted in a green rectangle: (A) PCL80, *S. aureus* 25923, and (B) PCL45, *S. aureus* 6538.

Nanofibrous materials are generally considered suitable materials for producing effective filtration membranes due to their well-defined, relatively homogeneous structure and modifiable pore size. A high retention rate of undesirable particles is necessary for ensuring their microbiological safety in all application areas, wound dressings, face masks, and food packaging, protecting patients/foods from external contamination foremost. Therefore, in the second part of the study, the ability of PCL nanomaterials to retain bacterial cells during filtration was tested and compared. To ensure robustness of the testing, two different cell concentrations were filtered. Retention was determined quantitatively by determination of retained CFU mL^–1^ and retention rate calculation (Table 3) with the statistical evaluation (Tables S2 and S8) and qualitatively by SEM (Figures 7 and 8).

**Table 3:** Evaluation of PCL’s Retention of Bacterial Cells of *S. aureus* 6538, *S. aureus* 25923, *E. coli* 8739, and *E. coli* 25922 Expressed as Determination of Filtered and Retained CFU ML^–1^ and Retention Rate (%)

material		PCL45				PCL80			
filtered cell concn		0.5 McF		1 McF		0.5 McF		1 McF	
average retention		retention [log(CFU mL^–1^)]	retention (%)	retention [log(CFU mL^–1^)]	retention (%)	retention [log(CFU mL^–1^)]	retention (%)	retention [log(CFU mL^–1^)]	retention (%)
bacterial strain	*E. coli* 25922	0.6 ± 0.1	75.0 ± 6.5	0.8 ± 0.0	85.4 ± 1.1	0.4 ± 0.2	54.5 ± 23.5	0.4 ± 0.1	62.9 ± 0.6
	*E. coli* 8739	1.1 ± 0.1	92.2 ± 1.4	1.1 ± 0.0	91.5 ± 0.8	0.5 ± 0.0	68.0 ± 3.3	0.1 ± 0.1	26.3 ± 14.9
	*S. aureus* 25923	5.8 ± 0.5	100.0 ± 0.0	6.7 ± 0.3	100.0 ± 0.0	0.9 ± 0.1	86.4 ± 4.6	0.5 ± 0.1	66.2 ± 8.4
	*S. aureus* 6538	7.0 ± 0.1	100.0 ± 0.0	7.0 ± 0.1	100.0 ± 0.0	0.8 ± 0.1	84.6 ± 4.8	0.8 ± 0.1	82.0 ± 3.8

The tested PCL nanomaterials retained a certain number of bacterial cells (Table 3). The retention rate did not differ among the two tested filtered cell concentrations (*p* > 0.05), which corresponds with the initial assumptions and proves the correctness of the measured data. Significant differences were noticed between both tested PCLs and among the retention of filtered bacterial species (*p* < 0.001). No difference was found in the retention of different strains of the same species (*p* > 0.05) (Table S8), which was expected based on similar cell sizes of strains within one species.

To be an effective barrier, materials should reflect the size of targeted filtered particles, necessitating membrane pores to be smaller than the particles themselves. Materials’ retention efficiency is thus directly dependent on nanofibers’ morphology, specifically fiber diameter, the characteristic distance between fibers, and surface density, along with the character and size of the filtered particles.^20^ Specifically, recent research points out that fiber diameter and surface density are the critical morphological parameters significantly influencing pore size and overall retention.^8,20,21^ In our study, PCL80, disponing with thicker fibers and higher characteristic distance δ between fibers, retained significantly fewer cells than PCL45 (*p* < 0.001). The retention of PCL80 ranged from 26–68% for *E. coli* strains and 66–86% for *S. aureus* strains, while PCL45 reached a higher retention rate ranging from 75–92% for *E. coli* strains and 100% for *S. aureus* strains (up to 7 log (CFU mL^–1^) retention) (Table 3). The results of the quantitative analysis were further supported by SEM analysis (Figures 7 and 8). Bacterial cells were retained in tangles of thin fibers, while no retention was noticed in areas of the nanomaterials, where thick fibers predominate (Figure 6). Consequently, the hypothesis H3: “PCL nanomaterials’ fiber diameter influences the retention of bacterial cells.” was substantiated.

For microbial filtrations, several polymers, mainly polyacrylonitrile (PAN) and PA, and their characteristics have been studied. Typically, polymers functionalized with antimicrobials or other active agents applicable to water treatment are used. For example, PAN functionalized with trimethylolpropane and triacrylate 1-(1-vinylimidazolium)ethyl-3-vinylimdazolium dibromide removed up to 99.9% of filtered *E. coli*,^22^ and similarly, PAN functionalized with chitosan and biochar removed up to 99% of coliform bacteria.^23^ In both cases, functionalization was considered to be the main factor increasing the effectiveness of PAN membranes. In contrast, research on nonfunctionalized materials, effective solely due to their structure, is rare in this field. Nonfunctionalized PA reduced *Saccharomyces cerevisiae*, *Flavobacterium johnsonidae*, and *Iodobacter fluviatilis* by up to 5 orders.^24^ Moreover, nonfunctionalized PA nanomaterials having fiber diameters ranging from 87 to 236 nm retained completely *S. aureus* cells, regardless of fiber diameter influence, in our previous study.^8^

While PCL is less common in these studies, knowledge of its ability to retain microbial cells is crucial for medical and biotechnological applications. Similar to the PAN, PCL filtration membranes are usually functionalized. Babu et al. reported that PCL membranes added with organoclay Cloisite 30B effectively retained filtered bacteria by qualitative observance.^25^ To the best of the author’s knowledge, no studies focusing on the analysis of the filtration properties of PCL based on their morphology have been published so far. Thus, we consider our results important to better understand the influence of PCL morphology on bacterial cell retention and highlight the need for further research. The development of PCL membranes capable of effectively retaining undesirable microbial cells solely on the basis of their morphology would increase the safety of used materials and allow a reduction in the amounts of antimicrobials required for materials functionalization. This approach opens the door for mitigating the risks associated with global antimicrobial resistance spread, a topic of considerable worldwide concern.

Further, as the size of filtered particles is besides materials morphology the second main parameter influencing retention, we investigated H4: “PCLs’ retention of *Staphylococcus* and *Escherichia* cells varies.” The results (Tables 3 and S8) clearly indicate that *E. coli* strains were retained by both PCLs much less than *S. aureus* strains (*p* < 0.001), confirming the acceptance of H4. *E. coli* forms rod-shaped cells with the size of 2.5 × 0.6 μm^2^, while *S. aureus* forms round-shaped cells ranging approximately from 0.5 to 1.5 μm. Based just on cell size, it could be incorrectly assumed that *E. coli* cells, being larger than *S. aureus* cells, should be retained more effectively. We presume that this is not the rule because of the ability of staphylococcal cells to assemble into specific grape-like structures, which was proven in our study. The staphylococcal cells gather immediately; thus, it is unavoidable to have the grape-like structures in the filtered suspension, and the larger structures are more easily retained than single cells. In follow-up studies, filtration analysis using fluorescently labeled cells could provide deeper insight into this problematic.^26^

The results not only support published studies highlighting the influence of nanofibers’ morphology on their retention but also provide insight into the behavior of PCL and also point out the markable differences between filtering of *E. coli* and *S. aureus* cells. Based on these results, PCL45 disponing with thin fibers and low characteristic distance δ between fibers can be considered a suitable material for further research. In general, we recommend producing PCL materials with fine structure, i.e., thin fibers and sufficient surface density, to ensure both good mechanical endurance and a dense network of fibers that guarantees impermeability for small particles.

## 4 Conclusion

In this study, two electrospun PCL nanomaterials differing in fiber diameter were tested in terms of their interactions with two strains of *E. coli* and two strains of *S. aureus*. First, the influence of PCL’s fiber diameter on biofilm formation was investigated. Biofilms formed easily on both PCL nanomaterials, yet no general conclusion regarding the influence of fiber diameter on biofilm formation was established. While fiber diameter affected the biofilm formation of one of the tested bacterial strains, *E. coli* 8739, it did not impact the biofilm formation of the other three tested strains. However, the fiber diameter influenced the structure of the biofilms formed by all the four tested bacterial strains; in PCL45, having thin fibers (376 ± 345 nm), thick uniform biofilm layers were formed preferably on the surface, while in PCL80, having thick fibers (945 ± 671 nm), smaller clusters formed preferably inside the structure. Further, the influence of PCL’s fiber diameter on the retention of bacterial cells was confirmed. PCL45, reaching up to 7 log (CFU mL^–1^) of staphylococcal cells (100% retention), retained significantly more cells than PCL80. *S. aureus* cells were in general retained by both the PCL nanomaterials more than *E. coli* cells, probably due to the tendency of staphylococcal cells to aggregate into larger, grape-like structures. The overall results confirm the influence of PCLs’ fiber diameter on the interactions with bacteria, indicating PCL45’s potential for further research and highlighting the differences in behavior of different strains of one species. We believe that these findings may accelerate the development of microbiologically safe nanomaterials applicable as wound dressings, face masks, or food packaging.

## Data Availability

Raw data from XPS measurement are available at DOI: 10.5281/zenodo.10955901.

## Supporting Information Available

Estimation of characteristic distances between fibers (methodology, parameters, results); data normality verification (Tables S1 and S2); wettability of the materials (Figure S1); biofilm formation by *E. coli* 8739, *E. coli* 25922, *S. aureus* 6538, and *S. aureus* 25923 on PCL nanomaterials PCL45 and PCL80 and control (PS) expressed as determination of CFU mL^–1^ and biofilm formation rate compared to control (%) (Table S3); statistical evaluation of data of biofilm formation (Tables S3–S7); statistical evaluation of data of bacterial cell retention (Table S3) (PDF)
