# Supplementary material for: Influence of Fiber Diameter of Polycaprolactone Nanofibrous Materials on Biofilm Formation and Retention of Bacterial Cells
Source: ACS Appl Mater Interfaces. 2024 May 8;16(20):25813–24. doi: 10.1021/acsami.4c03642 (PMC11129108; doi:10.1021/acsami.4c03642)
Supplement: Supplementary file 1 — am4c03642_si_001.pdf [file am4c03642_si_001.pdf]

SUPPORTING INFORMATION

Influence of Fiber Diameter of Polycaprolactone Nanofibrous Materials on Biofilm Formation and Retention of Bacterial Cells

Simona Lencova[1*], Marta Stindlova[1], Kristyna Havlickova[2], Vera Jencova[2], Vaclav Peroutka[1], Katerina Navratilova[1], Kamila Zdenkova[1], Hana Stiborova[1], Sarka Hauzerova[2], Eva Kuzelova Kostakova[2], Ondrej Jankovsky[3], Pavel Kejzlar[4], David Lukas[2], Katerina Demnerova[1]

[1] Department of Biochemistry and Microbiology, University of Chemistry and Technology, Prague, Czech Republic

[2] Department of Chemistry, Faculty of Science, Humanities and Education, Technical University of Liberec, Liberec, Czech Republic

[3] Department of Inorganic Chemistry, University of Chemistry and Technology Prague, Czech Republic

[4] Department of Advanced Materials, Institute for Nanomaterials, Advanced Technologies and Innovation, Technical University of Liberec, Liberec, Czech Republic

[*]corresponding author: Simona Lencova, lencovas@vscht.cz

Estimation of characteristic distances between fibers

Methodology:

In the simulations of highly porous planar fibrous material it is often used the approximate analytical expression for the average pores size δ, that obtained from the model of ordered cylindrical fibres (Lurie 2017), i.e., $\delta = \frac{\pi\, d}{4V_V}$. The average pores size has here also a meaning of a characteristic distance between fibres creating a pore in planar fibre process. Parameter d is the fibre diameter an V_V denotes the volume fraction of fibres. This approach motivates for characteristic distance estimation using the formula $\delta = \frac{1}{L_A}$, where L_A is the area density of the fibre process, i.e., the total fibre length in a unitary area. The density L_A is estimated here using the Buffon's needle problem (Stoyan 1995) as $L_A = \frac{\pi}{2} I_N$, where I_N is a length density of crossings between a testing line with a fibrous system, i.e., the number of crossings per a unitary length of the testing line. Therefore, we estimate here the characteristic intra-fiber distance δ as $\delta = \frac{2\,1}{\pi I_N}$.

Parameter:

L … line length

N … the number of intersections of the line with fibers

N_L … the number of intersections of the line and fibers per unit length of the line

δ … estimate of the characteristic distance between fibers

Results:

Characteristic distance δ in the nanofibrous sample PCL80 is estimated as 1,07 μm $\pm$ 0,08 μm and as 0,56 μm $\pm$ 0,1 μm for the sample PCL45.

References:

- S.A. Lurie, Y.O. Solyaev, D.V. Lizunova, L.N. Rabinskiy, V.M. Bouznik, O. MenshykovInternational Journal of Heat and Mass Transfer 109 (2017) 511–519. http://dx.doi.org/10.1016/j.ijheatmasstransfer.2017.02.015.
- D. Stoyan, W. S. Kendall, J. Mecke Stochastic, Geometry and Its Applications (Wiley Series in Probability & Statistics) 1995, ISBN-10: .:047195099

PCL80

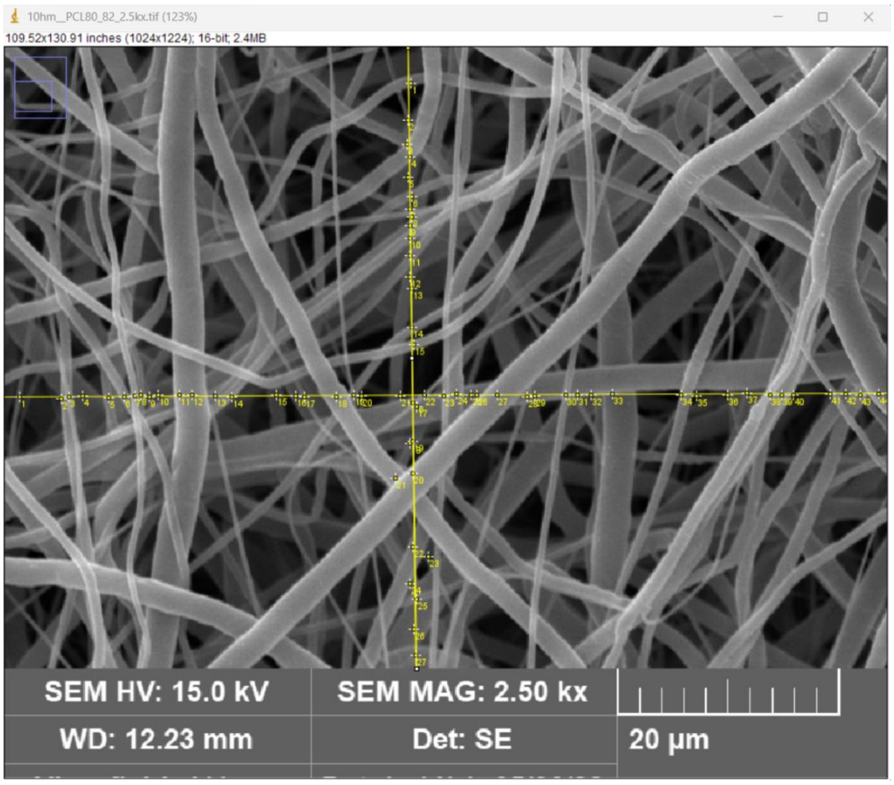

L= 78.931 μm, N=45, $N_L = \frac{45}{78.931} = 0.57012\ \mu m^{-1}$

L=55.620 μm, N=27, $N_L = \frac{27}{55.620} = 0.48543\ \mu m^{-1}$

Estimation of characteristic distances between fibers:

$$\delta = \frac{2\ 1}{\pi N_L}$$

PCL 80				
N	L	NL	δ	
45	78.931	0.570118	1.116678	
27	55.62	0.485437	1.311475	
29	59.947	0.483761	1.31602	
31	60.084	0.515944	1.233929	
26	49.146	0.529036	1.203394	
18	36.216	0.497018	1.280917	
			0.076241	STD [μm]
			1.07695	average [μm]

PCL45

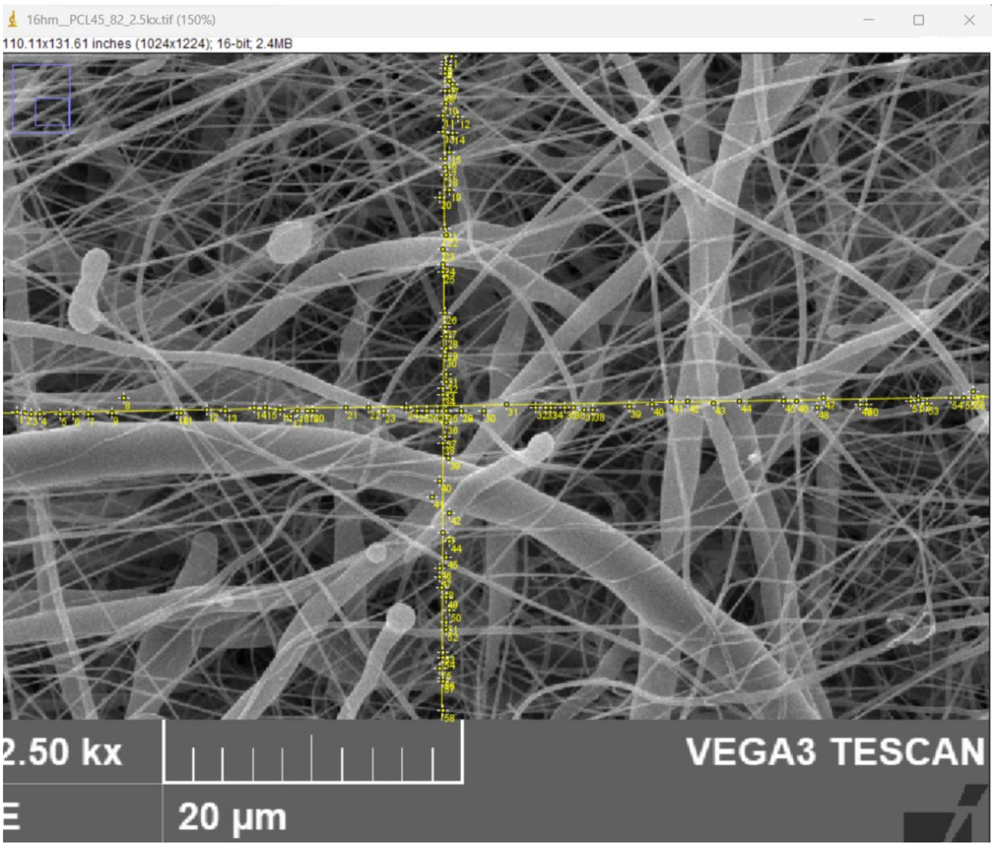

L=44.139 μm, N=58, , $N_L = \frac{58}{44.139} = 1.31403\ \mu m^{-1}$

L=65.707 μm, N=58, , $N_L = \frac{58}{65.707} = 0.88271\ \mu m^{-1}$

Estimation of characteristic distances between fibers:

PCL 45				
N	L [μm]	NL [μm^{-1}]	δ	
58	44.149	1.313733	0.484603	
58	65.707	0.882707	0.721235	
55	39.706	1.385181	0.459607	
42	39.836	1.054323	0.603837	
31	23.189	1.336841	0.476226	
33	32.703	1.009082	0.630909	
			0.10553	STD [μm]
			0.562736	average [μm]

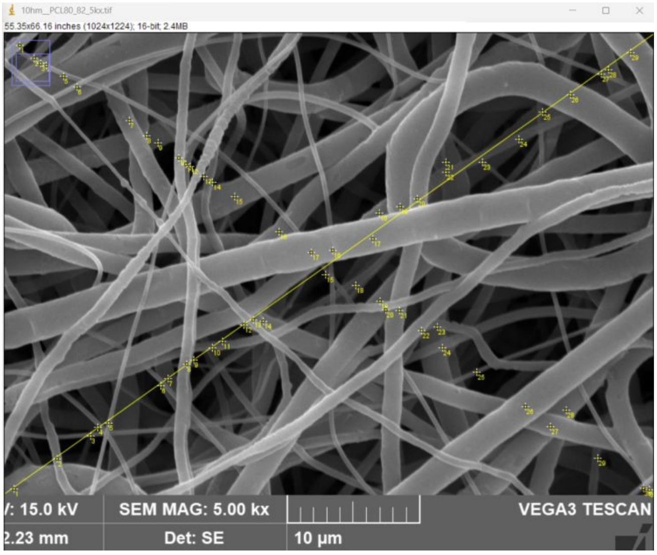
V: 15.0 kV
SEM MAG: 5.00 kx
VEGA3 TESCAN
2.23 mm
Det: SE
10 μm

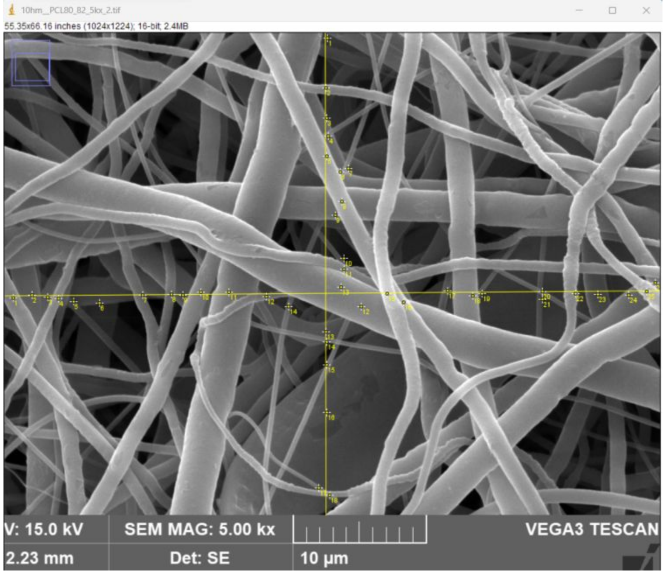
V: 15.0 kV
SEM MAG: 5.00 kx
VEGA3 TESCAN
2.23 mm
Det: SE
10 μm

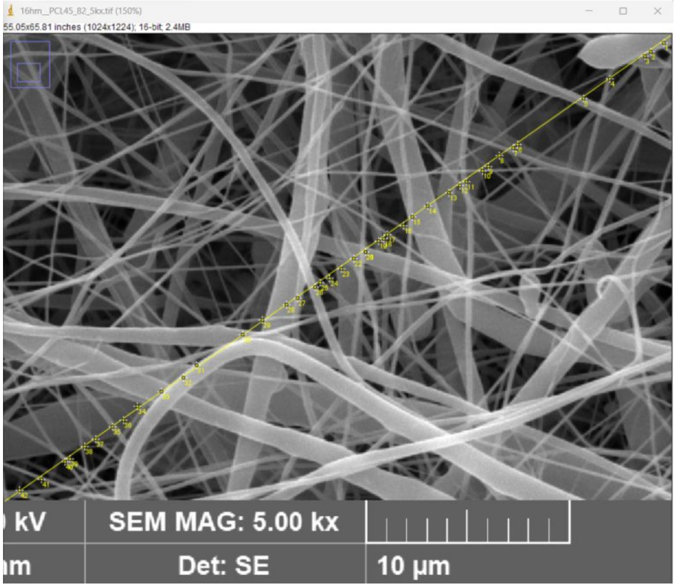
kV
SEM MAG: 5.00 kx
m
Det: SE
10 μm

Wettability of the materials

Figure S1: Water wicking into PCL45 and PCL80 nanofibrous materials over time. These are the average values of five individual measurements for each sample. The results represent the dynamic of the liquid wicking into the samples. The graphs presented are the average values for measurements of all samples from both types of materials. The graph can be divided into the first part where capillary forces are dominant and the second part where gravitational forces are already involved in the process against capillary forces. The break between the first and the second part occurs for PCL80 at a lower mass of the wicked liquid. This is probably due to the larger fibre diameters and hence the smaller specific surface area that the liquid gradually occupies in this type of test. Otherwise, it is not possible to see significant differences between the two materials with respect to their water wetting.

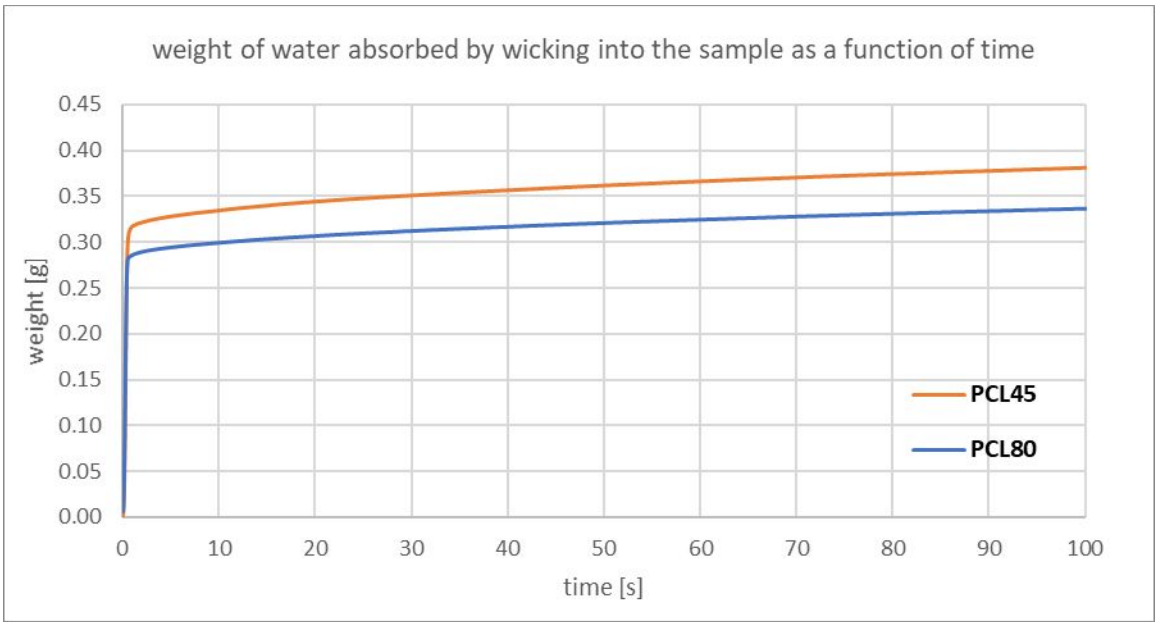

Data normality verification

Table S1: The results of biofilm growth measurements of all bacterial strains on the plate, PCL45 and PCL80, the Shapiro-Wilk test confirmed the normality of the distributions of the measured data (p-value > 0.05).

Shapiro-Wilk normality test	
strain_variation	p-value
SA25923_plate	0.6251
SA25923_PCL45	0.3084
SA25923_PCL80	0.4012
SA6538_plate	0.2032
SA6538_PCL45	0.1280
SA6538_PCL80	0.4872
EC25922_plate	0.5429
EC325922_PCL45	0.8224
EC25922_PCL80	0.1196
EC8739_plate	0.3731
EC8739_PCL45	0.5884
EC8739_PCL80	0.9639

Table S2: The results of filtration of all bacterial strains through PCL45 and PCL80, the Shapiro-Wilk test confirmed the normality of the distributions of the measured data (p-value > 0.05).

Shapiro-Wilk normality test	
strain_variation	p-value
SA25923_PCL45	0.6815
SA25923_PCL80	0.4180
SA6538_PCL45	0.8782
SA6538_PCL80	0.6976
EC25922_PCL45	0.8571
EC25922_PCL80	0.4371
EC8739_PCL45	0.6182
EC8739_PCL80	0.8936

Biofilm formation on PCL

Table S3: Biofilm formation by *E. coli* 8739, *E. coli* 25922, *S. aureus* 6538 and *S. aureus* 25923 on PCL nanomaterials PCL45 and PCL80 and control (PS) expressed as determination of CFU·ml^{-1} and biofilm formation rate compared to control (%).

Material		PS		PCL45		PCL80	
Average biofilm formation		CFU/cm^2	%	CFU/cm^2	%	CFU/cm^2	%
Bacterial strain	*E. coli* 25922	5.5E+07 ± 1.9E+07	100.0 ± 0.0	1.1E+07 ± 4.9E+06	19.5 ± 7.9	1.2E+07 ± 1.1E+07	19.7 ± 9.9
	E. coli 8739	6.7E+08 ± 5.5E+07	100.0 ± 0.0	3.9E+08 ± 1.1E+07	61.3 ± 42.4	1.1E+08 ± 1.2E+07	17.7 ± 15.1
	S. aureus 25923	5. 9E+07 ± 4.5E+07	100.0 ± 0.0	2.1E+07 ± 2.7E+07	29.8 ± 20.1	2.1E+07 ± 2.8E+07	37.6 ± 28.9
	S. aureus 6538	1.6E+08 ± 6.9E+07	100.0 ± 0.0	1.1E+07 ± 5.9E+06	8.5 ± 5.9	2.1E+07 ± 3.2E+07	13.1 ± 10.8

Table S4: Statistical evaluation of data - testing whether biofilm growth on PCL is significantly different from the control on the plate. The paired t-test method was used, the condition of normal data distribution was met and verified using the Shapiro-Wilk test (at α = 0.05).

Paired t-test				
strain_variation	control	t-test statistic value	p-value	mean differences between pairs
SA25923_PCL45	SA25923_plate	3.72	5.45E-03	1.33
SA25923_PCL80	SA25923_plate	3.75	5.29E-03	1.49
SA6538_PCL45	SA6538_plate	8.96	1.91E-05	1.20
SA6538_PCL80	SA6538_plate	5.70	4.56E-04	1.17
EC25922_PCL45	EC25922_plate	8.66	2.47E-05	0.72
EC25922_PCL80	EC25922_plate	3.64	6.84E-03	0.85
EC8739_PCL45	EC8739_plate	2.52	3.56E-02	0.36
EC8739_PCL80	EC8739_plate	7.64	8.91E-05	0.81

Table S5: Statistical evaluation of data – testing of difference in biofilm growth on PCL45 versus PCL80; the unpaired t-test method was used, the condition of normal data distribution was met and verified using the Shapiro-Wilk test (at α = 0.05).

Unpaired Two-Samples t-test			
strain_variation_1	strain_variation_2	p-value	mean differences between pairs
SA25923_PCL45	SA25923_PCL80	0.7645	0.1611
SA6538_PCL45	SA6538_PCL80	0.9056	-0.0256
EC25922_PCL45	EC25922_PCL80	0.5971	0.1300
EC8739_PCL45	EC8739_PCL80	0.0154	0.4532

Table S6: Statistical evaluation of data – testing of a significant difference in the growth of biofilm of *S. aureus* and *E. coli* on individual materials; the one-factor ANOVA method was used ($p < 0.05$ = biofilm formation differs significantly within the selected groups at the level of significance $\alpha = 0.05$), the condition of normal data distribution was met and verified using the Shapiro-Wilk test.

One way ANOVA		
	PCL45	PCL80
F_value	17.54	6.456
Pr (>F)	6.47E-07	1.67E-03
signif. code	***	**

Table S7: Statistical evaluation of data – testing for which particular strains does the biofilm formation differ. Multiple pairwise comparisons was performed to determine if the mean difference between specific pairs of a group is statistically significant. Since the ANOVA test is significant, we can calculate Tukey HSD (Tukey Honest Significant Differences) to perform multiple pairwise comparisons (group means) – a) for PCL45 and b) for PCL80. For both PCL45 and PCL80, the results of the TukeyHSD multiple comparison test show that there is a significant difference between the biofilm formation of EC8739 and EC25922, between SA25923 and EC8739, and between SA6538 and EC8739 at the given adjusted p-values (p adj). In general: on both PCL45 and PCL80 there is a significantly difference in EC8739 biofilm compared to the other strains. The differences in biofilm formation of the other tested isolates are not statistically different.

a)

PCL45		diff	p adj
EC8739	EC25922	1.4744	4.90E-05
SA25923	EC25922	-0.3889	0.5117
SA6538	EC25922	-0.0267	0.9997
SA25923	EC8739	-1.8633	9.00E-07
SA6538	EC8739	-1.5011	3.72E-05
SA6538	SA25923	0.3622	0.5702

b)

PCL80		diff	p adj
EC8739	EC25922	1.1513	0.0235
SA25923	EC25922	-0.4200	0.6660
SA6538	EC25922	0.1289	0.9849
SA25923	EC8739	-1.5713	9.67E-04
SA6538	EC8739	-1.0224	0.0432
SA6538	SA25923	0.5489	0.4269

PCLs retention of bacterial cells

Table S8: Statistical evaluation of data – testing of a significant difference in the retention of *S. aureus* (SA) and *E. coli* (EC) by PCL45 and PCL80; the one-factor ANOVA method was used ($p < 0.001$ = retention differs significantly within the selected groups at the level of significance $\alpha = 0.001$, marked as ***; $p > 0.05$ = retention does not differ significantly within the selected groups at the level of significance $\alpha = 0.05$, marked as -), the condition of normal data distribution was met and verified using the Shapiro-Wilk test.

One way ANOVA			
**Test_hypothesis**	**F_value**	**Pr (>F)**	**signif. code**
Retention of PCL45 and PCL80 differs.	26.19	5.91E-06	***
Retention of EC and SA by PCL45 differs.	19.86	1.98E-04	***
Retention of EC and SA by PCL80 differs.	19.86	1.98E-04	***
Retention of different filtered concentrations of EC by PCL45 differs.	1.77	2.00E-01	-
Retention of different filtered concentrations of EC by PCL80 differs.	1.76	2.15E-01	-
Retention of different filtered concentrations of SA by PCL45 differs.	1.85	2.04E-01	-
Retention of different filtered concentrations of SA by PCL80 differs.	6.16	3.25E-02	-
PCL45´s retention of different EC strains does not differ.	0.86	3.76E-01	-
PCL45´s retention of different SA strains does not differ.	7.31	2.22E-02	-
PCL80´s retention of different EC strains does not differ.	0.79	3.96E-01	-
PCL80´s retention of different SA strains does not differ.	0.76	4.04E-01	-
